# Effects of secretome derived from macrophages exposed to calcium oxalate crystals on renal fibroblast activation

**DOI:** 10.1038/s42003-021-02479-2

**Published:** 2021-08-11

**Authors:** Sunisa Yoodee, Chadanat Noonin, Kanyarat Sueksakit, Rattiyaporn Kanlaya, Sakdithep Chaiyarit, Paleerath Peerapen, Visith Thongboonkerd

**Affiliations:** Medical Proteomics Unit, Office for Research and Development, Faculty of Medicine Siriraj Hospital, Mahidol University, Bangkok, Thailand

**Keywords:** Monocytes and macrophages, Proteomics, Proteome, Proteomics, Mechanisms of disease

## Abstract

The association between kidney stone disease and renal fibrosis has been widely explored in recent years but its underlying mechanisms remain far from complete understanding. Using label-free quantitative proteomics (nanoLC-ESI-LTQ-Orbitrap MS/MS), this study identified 23 significantly altered secreted proteins from calcium oxalate monohydrate (COM)-exposed macrophages (COM-MP) compared with control macrophages (Ctrl-MP) secretome. Functional annotation and protein-protein interactions network analysis revealed that these altered secreted proteins were involved mainly in inflammatory response and fibroblast activation. BHK-21 renal fibroblasts treated with COM-MP secretome had more spindle-shaped morphology with greater spindle index. Immunofluorescence study and gelatin zymography revealed increased levels of fibroblast activation markers (α-smooth muscle actin and F-actin) and fibrotic factors (fibronectin and matrix metalloproteinase-9 and -2) in the COM-MP secretome-treated fibroblasts. Our findings indicate that proteins secreted from macrophages exposed to COM crystals induce renal fibroblast activation and may play important roles in renal fibrogenesis in kidney stone disease.

## Abstract (web summary)

Yoodee et al use label-free quantitative proteomics (nanoLC-ESI-LTQ-Orbitrap MS/MS) to identify 23 significantly altered secreted proteins from calcium oxalate monohydrate (COM)-exposed macrophages secretome. They demonstrate that proteins secreted from macrophages exposed to COM crystals induce renal fibroblast activation and may play important roles in renal fibrogenesis in kidney stone disease.

## Introduction

Calcium oxalate (CaOx) monohydrate (COM) is a major inorganic component in kidney stones^1–3^. Idiopathic CaOx kidney stone is formed mainly by Randall’s plaque mechanism, in which supersaturated calcium phosphate starts to deposit at subepithelial or interstitial locale followed by Randall’s plaque formation^4–6^. The main originating site for Randall’s plaque formation is thin ascending limb of Henle’s loop^7^. The plaque at papilla’s tip can then erode into renal pelvis, where CaOx is frequently supersaturated, and serves as the nidus for CaOx stone formation^7–9^. An alternative mechanism describes a possibility of paracellular translocation of CaOx crystals to the renal interstitium through the disrupted tight junctions or neocrystallization after the degraded intracellular crystals generate free calcium and oxalate ions that pass through basolateral membranes of renal tubular cells^8,10–12^.

The interstitial CaOx crystal deposition can trigger tissue injury and induce macrophage recruitment^13^. Previous studies in both humans and animal models have shown accumulation of macrophages around the CaOx crystals, suggesting their roles in eliminating the crystals by phagocytosis^14–16^. The CaOx crystal-exposed macrophages can also secrete various proinflammatory cytokines, e.g., interleukin-1β (IL-1β), IL-6, tumor necrosis factor-α (TNF-α)^17–21^. These proinflammatory cytokines have been shown to promote secretion of other cytokines and chemokines from both immune and non-immune cells, thereby enhancing leukocyte recruitment and inflammatory response^22,23^.

Fibrosis is a disorder that can occur in several various organs characterized by aberrant, excessive production and accumulation of extracellular matrix (ECM) in the tissues^24–26^. Fibroblast and myofibroblast are the main effector cells responsible for tissue fibrotic changes and overproduction of ECM proteins, e.g., types I and III collagens, fibronectin^27^. In the kidney, myofibroblast can be differentiated from various cells, but its main source (approximately 50%) seems to be residential fibroblast in renal interstitium^28–30^. Transformation of fibroblast into its activated form, myofibroblast, is induced mainly by transforming growth factor-β (TGF-β) secreted from inflammatory cells in the inflammatory milieu via various signaling pathways^31–34^.

Renal fibrosis is frequently observed in histopathological study of kidney tissues from the stone formers^35–37^. Nevertheless, mechanisms underlying kidney stone-associated renal fibrosis remain largely unknown. In this study, we explored the roles for proteins secreted from macrophages exposed to COM crystals in transformation of renal fibroblast into its active form, myofibroblast. Comparative analysis of secretome (a set of secretory proteins) derived from the serum-free culture supernatant of the COM-exposed macrophages (COM-MP) versus that of the control macrophages (Ctrl-MP) was performed by label-free quantitative proteomics using nanoLC-ESI-LTQ-Orbitrap MS/MS. Functional annotation and protein-protein interactions network analysis were done to address functional significance of the significantly altered secreted proteins. Finally, effects of the COM-MP secretome on renal fibroblast activation were addressed compared with the Ctrl-MP secretome using various assays.

## Results

### Defining the optimal time-point for analysis of secretome derived from COM-exposed macrophages (COM-MP)

Prior to sophisticated investigations, the study model must be carefully defined. To be able to examine the “secretome” (a set of secretory proteins), serum or other source of exogenous proteins must be omitted from the culture medium. Without serum supplementation, the cells cannot maintain their healthy state so long because growth factors and nutrients rich in the serum are essential for maintaining the normal cell physiology and proliferative cycle. While secretion of the proteins from the cells are usually time-dependent, prolonging culture in the serum-free condition would be accompanied with cytotoxicity and cell death, which can easily interfere with the secretome analysis (because the toxic or dead cells can release cellular proteins from disruption, not by secretion). On the other hand, secretion of proteins is too trivial when the cells are incubated in the serum-free medium only briefly.

**Fig. 1:**
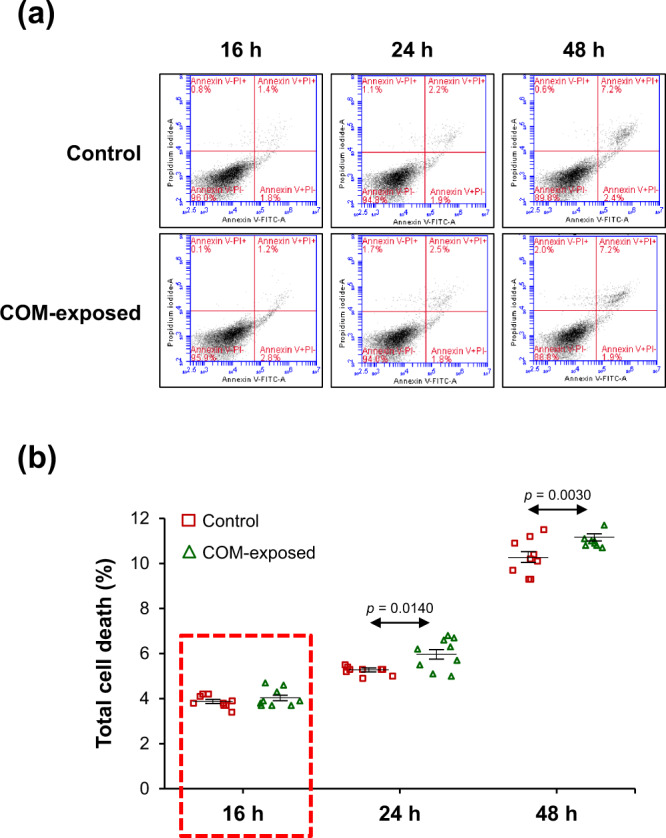
Cell death assay by flow cytometry with annexin V/propidium iodide co-staining. Macrophages were incubated in serum-free medium with or without 100 µg/ml COM crystals for 16, 24, and 48 h. The cells were then collected and co-stained with annexin V and propidium iodide followed by flow cytometric analysis. **a** Scatter plots showing fluorescence signal of FITC-conjugated annexin V (X-axis) and propidium iodide (Y-axis). **b** Quantitative analysis of cell death calculated by using Formula 1. The data were obtained from three independent experiments using different biological replicates and the bars indicate mean ± SEM (all the source data are presented in Supplementary Data 1). Red-dashed rectangle indicates the optimal time-point that was chosen for all subsequent experiments.

To define the optimal time-point for secretome collection for subsequent analyses, the U937-derived macrophages were incubated in the serum-free medium with or without 100 µg/ml COM crystals for up to 48 h. Flow cytometric analysis with annexin V/propidium iodide co-staining was performed to evaluate cell death at 16, 24, and 48-h post-incubation. Quantitative analysis revealed that the COM-MP at 16-h post-incubation had no significant increase in cell death compared with the control macrophages (Ctrl-MP) (Fig. 1 and Supplementary Data 1). On the other hand, cell death significantly increased when macrophages were incubated with COM crystals for 24 and 48 h (Fig. 1 and Supplementary Data 1). Therefore, we selected 16-h as the optimal incubation period to evaluate the macrophage secretome upon COM crystal exposure throughout the study.

### Label-free quantitative proteomics analysis of differentially secreted proteins from COM-MP vs. Ctrl-MP

**Fig. 2:**
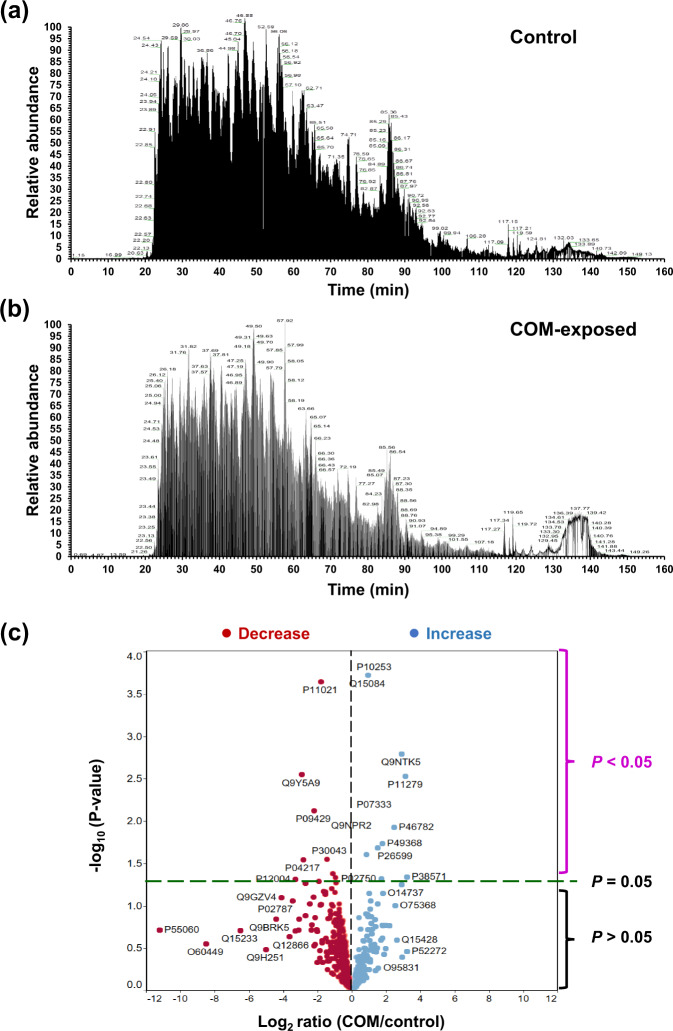
Label-free quantitative proteomics analysis of secretome derived from macrophages using nanoLC-ESI-LTQ-Orbitrap MS/MS. **a** Representative total ion chromatogram (TIC) of secretome derived from the control macrophages. **b** Representative TIC of secretome derived from the COM-exposed macrophages. **c** Volcano plot to demonstrate the relationship between differential ratios (COM/control) and *P*-values of all 598 identified proteins. All the source data have been deposited to the ProteomeXchange Consortium (http://www.proteomexchange.org/) via the PRIDE (https://www.ebi.ac.uk/pride/) partner repository with the dataset identifier PXD027039 and 10.6019/PXD027039. Proteins with *P* < 0.05 were considered as significantly altered secreted proteins.

Macrophages were incubated in serum-free medium with or without 100 µg/ml COM crystals for 16 h. The culture supernatants were collected, clarified and subjected to label-free quantitative proteomics analysis using three independent biological samples per group, and each sample was run in technical triplicates. Therefore, nine mass spectral profiles were obtained from each group for reliable quantitative analysis. Total ion chromatogram (TIC) representing the chromatographic profile (retention time vs. relative abundance) of proteins was obtained from each sample. The TIC profiles of Ctrl-MP and COM-MP secretomes were similar except for an area at 130–150 min retention time and few other locales where some differences between the two groups were quite obvious (Fig. 2a and b). Using nanoLC-ESI-LTQ-Orbitrap tandem mass spectrometry (MS/MS), a total of 598 proteins were identified from the macrophage secretome. The relationship between their abundance ratios of the two groups (COM/Control) and *P*-values was analyzed by a volcano plot (Fig. 2c). Among these, quantitative and statistical analyses revealed significant changes in levels of 23 proteins in secretome derived from the COM-MP compared with that derived from the Ctrl-MP (Table 1).

**Table 1:** Summary of significantly altered proteins in COM-exposed macrophage secretome identified by nanoLC-ESI-LTQ-Orbitrap MS/MS.

Protein name	Swiss-Prot ID	Intensity (x10^3^ A.U.) (Mean ± SEM)		Ratio	*P-*value
		Control	COM-exposed	(COM/Control)	
40 S ribosomal protein S5	P46782	1068 ± 744	5881 ± 507	5.51	0.0118
Adenylyl cyclase-associated protein 1	Q01518	21326 ± 4921	37953 ± 1522	1.78	0.0248
Alpha-1B-glycoprotein	P04217	63867 ± 22042	8957 ± 1990	0.14	0.0287
Cathepsin Z	Q9UBR2	118146 ± 27416	42883 ± 4964	0.36	0.0282
Endoplasmic reticulum chaperone BiP	P11021	68106 ± 9978	19309 ± 875	0.28	0.0002
Flavin reductase (NADPH)	P30043	0 ± 0	5862 ± 774	#DIV/0!	0.0226
Guanine nucleotide-binding protein G(I)/G(S)/G(T) subunit beta-1	P62873	0 ± 0	6466 ± 852	#DIV/0!	0.0224
High mobility group protein B1	P09429	110802 ± 28122	23932 ± 1311	0.22	0.0075
InaD-like protein	Q8NI35	1111533 ± 226328	563389 ± 38071	0.51	0.0461
Inter-alpha-trypsin inhibitor heavy chain H2	P19823	151029 ± 35882	70060 ± 2323	0.46	0.0416
Leucine-rich alpha-2-glycoprotein	P02750	4250 ± 2872	13809 ± 1140	3.25	0.0481
Lysosomal acid lipase/cholesteryl ester hydrolase	P38571	1117 ± 1117	10383 ± 1373	9.30	0.0453
Lysosomal alpha-glucosidase	P10253	0 ± 0	6470 ± 1314	#DIV/0!	0.0002
Lysosome-associated membrane glycoprotein 1	P11279	366 ± 366	3206 ± 241	8.76	0.0029
Macrophage colony-stimulating factor 1 receptor	P07333	0 ± 0	23119 ± 2459	#DIV/0!	0.0064
Obg-like ATPase 1	Q9NTK5	1188 ± 788	8839 ± 619	7.44	0.0016
Polypyrimidine tract-binding protein 1	P26599	4541 ± 1966	12817 ± 852	2.82	0.0207
Proliferating cell nuclear antigen	P12004	34503 ± 14300	3524 ± 824	0.10	0.0486
Protein disulfide-isomerase A6	Q15084	5607 ± 848	10622 ± 201	1.89	0.0002
Semaphorin-4B	Q9NPR2	0 ± 0	549 ± 62	#DIV/0!	0.0090
Splicing factor, proline- and glutamine-rich	P23246	0 ± 0	4088 ± 569	#DIV/0!	0.0293
T-complex protein 1 subunit gamma	P49368	2181 ± 1115	7487 ± 561	3.43	0.0183
YTH domain-containing family protein 2	Q9Y5A9	55802 ± 13271	7254 ± 1229	0.13	0.0028

*A.U.* arbitrary unit, *#DIV/0!* divided by zero (or newly present).

### Functional annotation and protein–protein interactions network analysis of the significantly altered secreted proteins

**Fig. 3:**
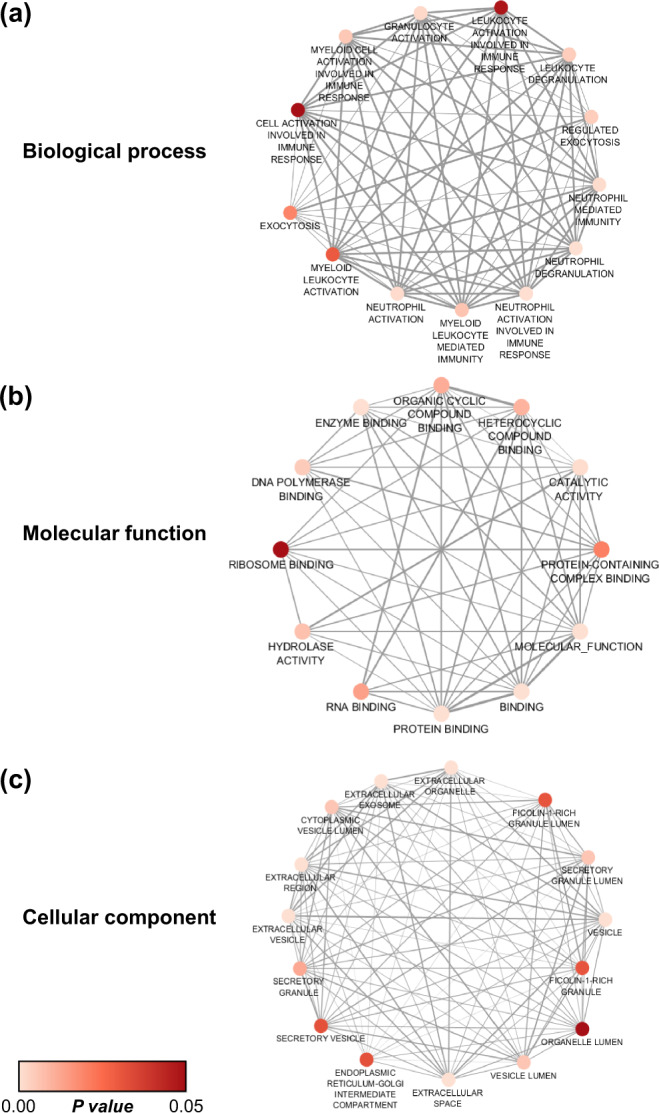
Functional annotation of the significantly altered secreted proteins. Functional enrichment analyses of all significantly altered secreted proteins identified from label-free quantitative proteomics using nanoLC-ESI-LTQ-Orbitrap MS/MS (as detailed in Fig. 2 and Table 1) were performed in g:Profiler web server using g:GOSt tool (https://biit.cs.ut.ee/gprofiler/gost). Their biological processes (**a**), molecular functions (**b**), and cellular components (**c**) were visualized using Cytoscape (version 3.7.2).

**Fig. 4:**
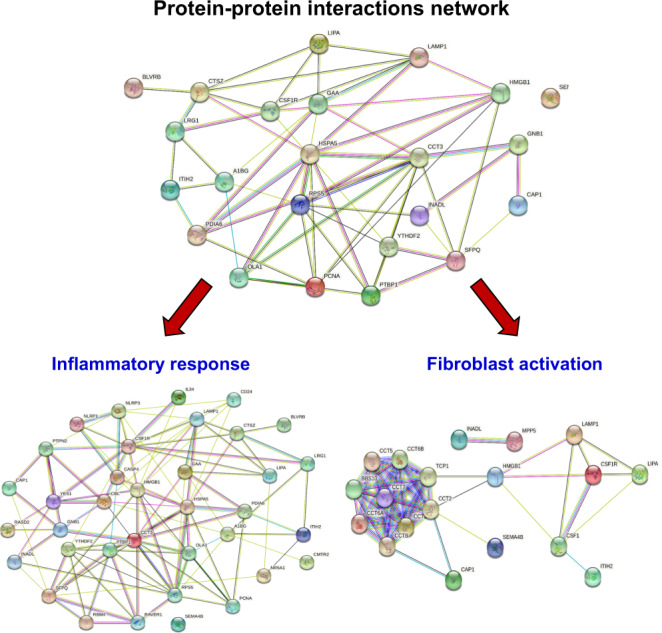
Protein–protein interactions network analysis. Protein–protein interactions network of all significantly altered secreted proteins from secretome of the COM-exposed vs. control macrophages were analyzed by STRING tool (version 11.0) (www.string-db.org). The data indicated that the significantly altered secreted proteins were involved mainly in “inflammatory response” and “fibroblast activation”.

Functional enrichment analysis of all significantly altered proteins were performed using g:GOSt tool in g:Profiler web server to retrieve their biological processes, molecular functions, and original cellular components. Cytoscape software was then used to visualize the interactions of their biological processes (Fig. 3a), molecular functions (Fig. 3b), and original cellular components (Fig. 3c). From these, the main biological process was immune response, the main molecular function was protein binding, and the main original cellular component was extracellular vesicle/exosome (Fig. 3). In addition, protein-protein interactions network was analyzed using STRING tool version 11.0. The data suggested that these differentially secreted proteins were involved mainly in “inflammatory response” and “fibroblast activation” (Fig. 4). Therefore, we further addressed effects of the COM-MP secretome on renal fibroblast activation.

### Effects of the COM-MP secretome on renal fibroblast morphology and spindle index

**Fig. 5:**
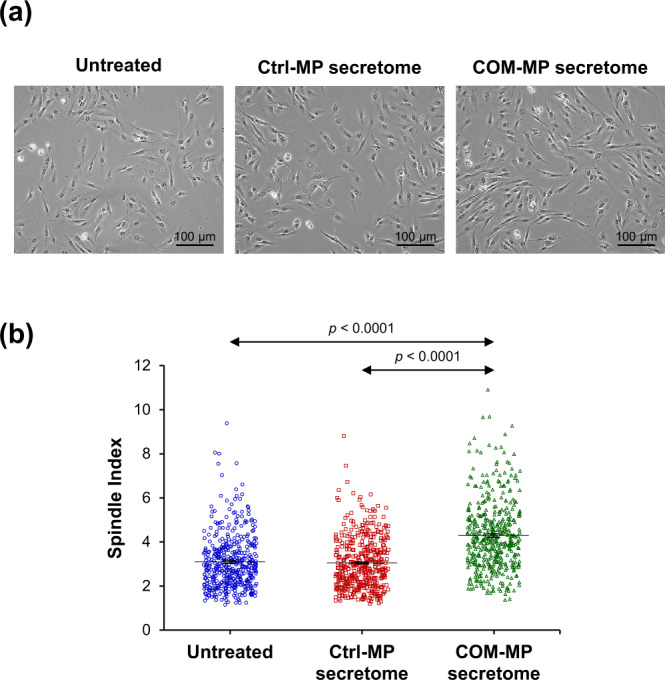
Effects of secretome derived from COM-exposed macrophages (COM-MP) on morphology and spindle index of BHK-21 renal fibroblasts. BHK-21 cells were incubated in fresh serum-free medium without any treatment (untreated control) or in fresh serum-free medium mixed 1:1 (v/v) with secretome derived from the control macrophages (Ctrl-MP secretome) or COM-MP secretome for 24 h. **a** Morphology of the BHK-21 renal fibroblasts. **b** Their spindle index was calculated from at least 100 cells in ≥10 random high-power fields (HPFs) for each sample using Formula 2. The data were obtained from three independent experiments using different biological replicates and the bars indicate mean ± SEM (all the source data are presented in Supplementary Data 2).

BHK-21 renal fibroblasts were cultured in fresh culture medium mixed 1:1 (v/v) with culture supernatant of the Ctrl-MP or the COM-MP, whereas those cultured in only fresh medium served as the untreated controls. After 24-h incubation, morphology of the Ctrl-MP secretome-treated fibroblasts and the untreated controls looked similar, whereas the COM-MP secretome-treated cells were elongated and had more spindle-shaped morphology (Fig. 5a). In concordance, the spindle index of the COM-MP secretome-treated fibroblasts significantly increased compared with that of the Ctrl-MP secretome-treated cells and the untreated controls (Fig. 5b and Supplementary Data 2).

### Effects of the COM-MP secretome on renal fibroblast activation markers

**Fig. 6:**
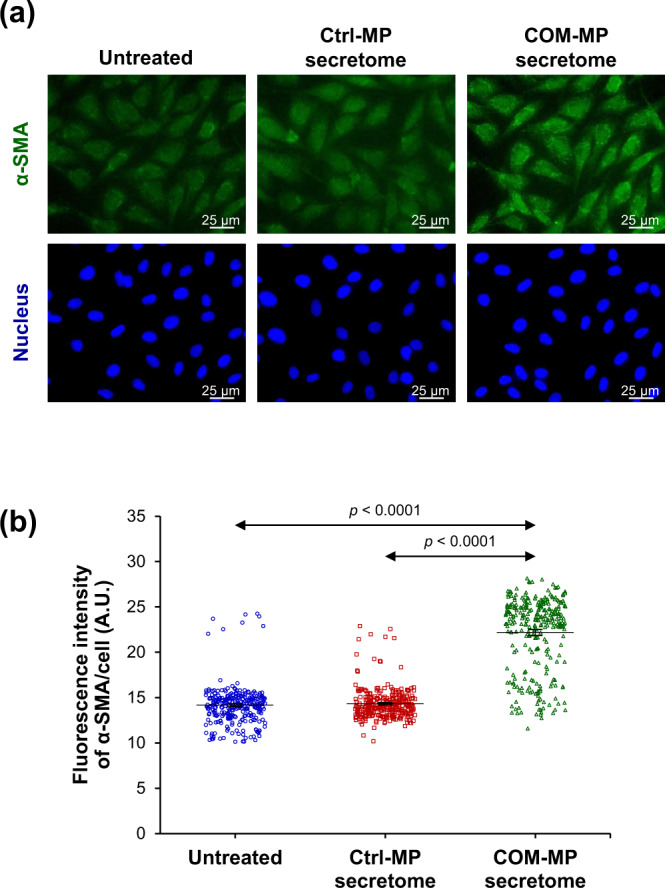
Effects of COM-MP secretome on α-SMA level in BHK-21 renal fibroblasts. BHK-21 cells were incubated in fresh serum-free medium without any treatment (untreated control) or in fresh serum-free medium mixed 1:1 (v/v) with Ctrl-MP secretome or COM-MP secretome for 24 h. **a** Immunofluorescence staining of α-SMA was done using mouse monoclonal anti-α-SMA as a primary antibody, whereas secondary antibody was conjugated with Alexa Flour 488. Nuclei were stained in blue using Hoechst dye. **b** Quantitative analysis of fluorescence intensity of α-SMA was obtained from at least 100 cells in ≥10 random HPFs for each sample using NIS-Elements D V.4.11 (Nikon). The data were obtained from three independent experiments using different biological replicates and the bars indicate mean ± SEM (all the source data are presented in Supplementary Data 3). A.U. = arbitrary unit.

**Fig. 7:**
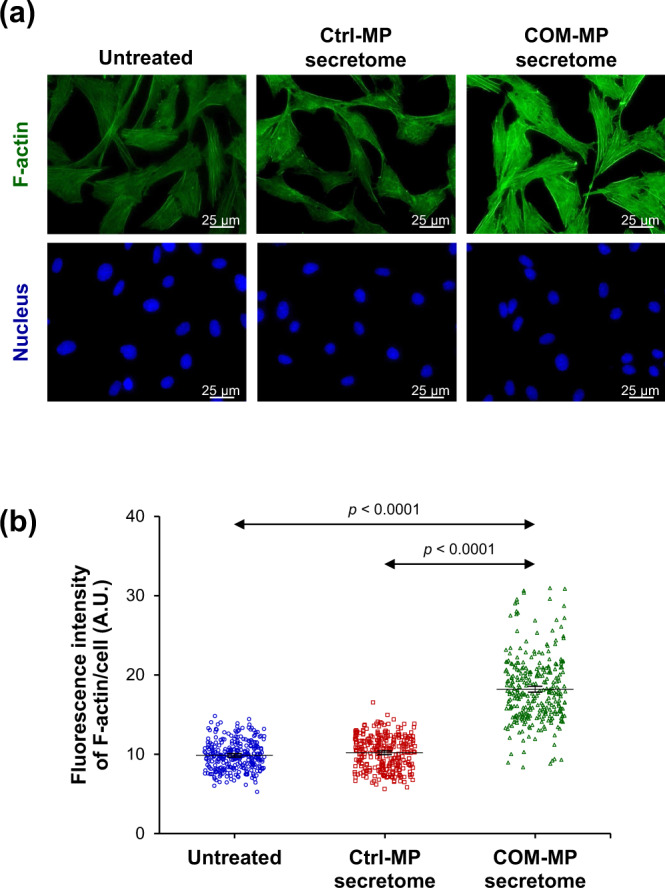
Effects of COM-MP secretome on F-actin level in BHK-21 renal fibroblasts. BHK-21 cells were incubated in fresh serum-free medium without any treatment (untreated control) or in fresh serum-free medium mixed 1:1 (v/v) with Ctrl-MP secretome or COM-MP secretome for 24 h. **a** Immunofluorescence staining of F-actin using Oregon Green 488-conjugated phalloidin. Nuclei were stained in blue using Hoechst dye. **b** Quantitative analysis of fluorescence intensity of F-actin was obtained from at least 100 cells in ≥10 random HPFs for each sample using NIS-Elements D V.4.11 (Nikon). The data were obtained from three independent experiments using different biological replicates and the bars indicate mean ± SEM (all the source data are presented in Supplementary Data 4). A.U. = arbitrary unit.

Since renal fibroblasts treated with the COM-MP secretome had more spindle-shaped morphology and greater spindle index, which are the features of fibroblast activation, expression of renal fibroblast activation markers was examined. Immunofluorescence stainings and quantitative analyses revealed that levels of α-SMA (Fig. 6 and Supplementary Data 3) and actin stress fiber (F-actin) (Fig. 7 and Supplementary Data 4), both of which are the fibroblast activation markers, significantly increased in the COM-MP secretome-treated fibroblasts compared with the Ctrl-MP secretome-treated cells and the untreated controls.

### Effects of the COM-MP secretome on production and secretion of fibrotic factors from the activated renal myofibroblasts

**Fig. 8:**
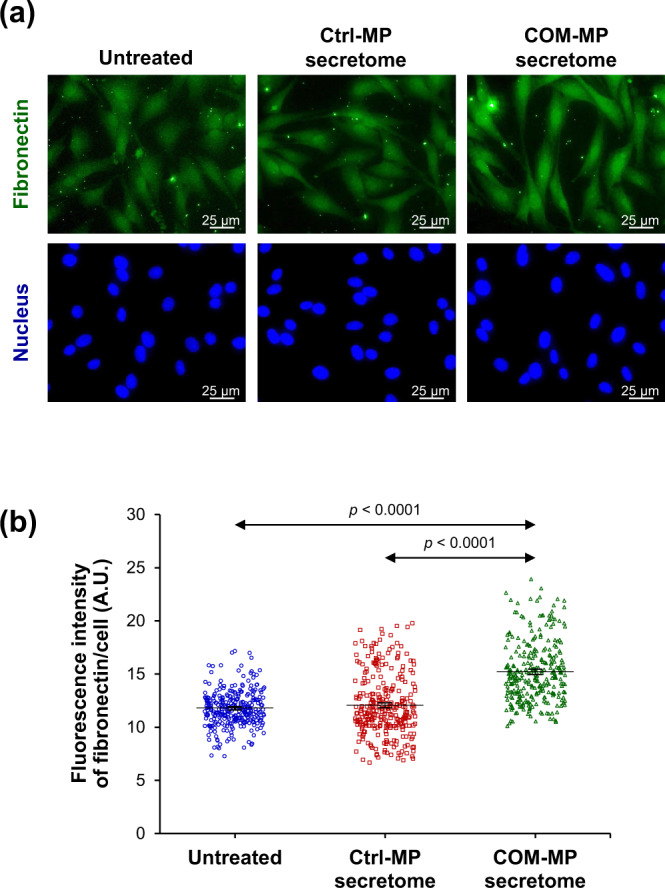
Effects of COM-MP secretome on fibronectin level in BHK-21 renal fibroblasts. BHK-21 cells were incubated in fresh serum-free medium without any treatment (untreated control) or in fresh serum-free medium mixed 1:1 (v/v) with Ctrl-MP secretome or COM-MP secretome for 24 h. **a** Immunofluorescence staining of fibronectin was done using mouse monoclonal anti-fibronectin as a primary antibody, whereas secondary antibody was conjugated with Alexa Flour 488. Nuclei were stained in blue using Hoechst dye. **b** Quantitative analysis of fluorescence intensity of fibronectin was obtained from at least 100 cells in ≥10 random HPFs for each sample using NIS-Elements D V.4.11 (Nikon). The data were obtained from three independent experiments using different biological replicates and the bars indicate mean ± SEM (all the source data are presented in Supplementary Data 5). A.U. = arbitrary unit.

**Fig. 9:**
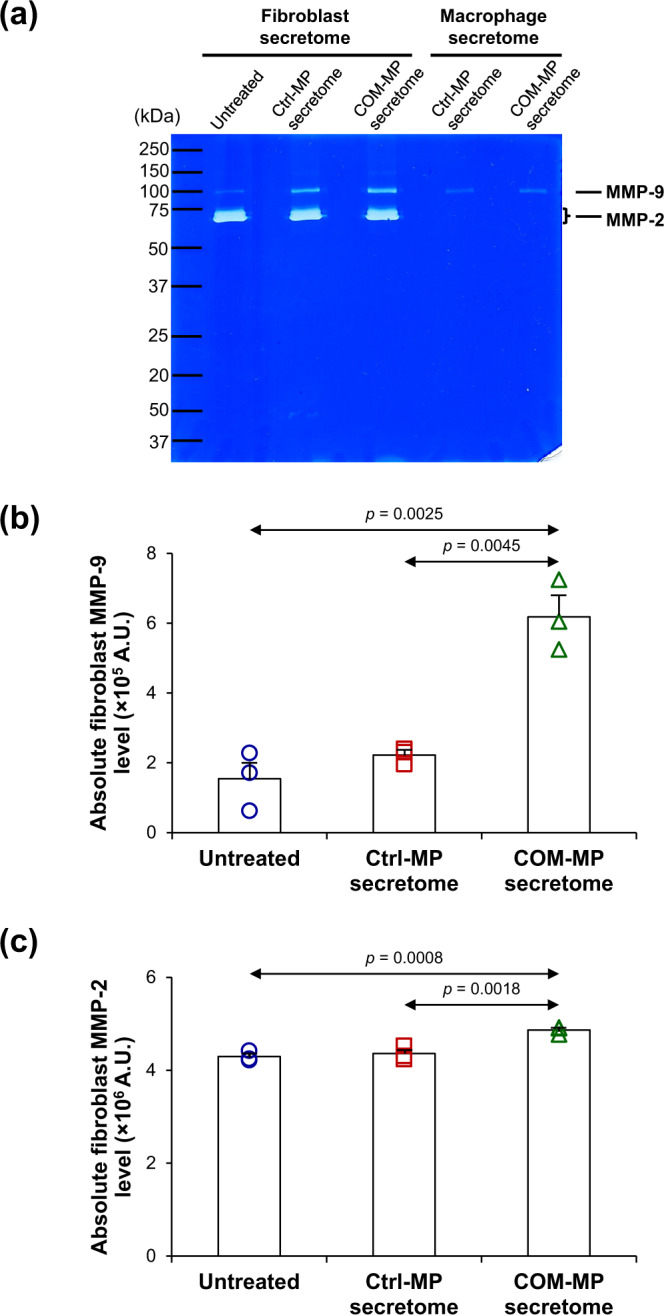
Effects of COM-MP secretome on MMPs activities. BHK-21 cells were incubated in fresh serum-free medium without any treatment (untreated control) or in fresh serum-free medium mixed 1:1 (v/v) with Ctrl-MP secretome or COM-MP secretome for 24 h. Thereafter, the clarified culture supernatant was subjected to gelatin zymogram assay. **a** MMP-9 and MMP-2 appeared as transparent bands at approximately 90 kDa and 65-70 kDa, respectively. **b**, **c** Absolute fibroblast levels of MMP-9 and MMP–2, respectively, were calculated using Formula 3. The data were obtained from three independent experiments using different biological replicates and the bars indicate mean ± SEM (all the source data are presented in Supplementary Data 6 and Supplementary Data 7, respectively). A.U. = arbitrary unit.

To investigate effects of the COM-MP secretome on fibrogenesis, expression and secretion of fibrotic factors, including fibronectin and MMPs, were evaluated. Level of fibronectin, which is one of the ECM components, was significantly greater in the COM-MP secretome-treated fibroblasts compared with the untreated and Ctrl-MP secretome-treated cells (Fig. 8 and Supplementary Data 5). For MMPs, gelatin zymogram assay revealed that both MMP-9 and MMP-2 were present as the clear bands at approximately 90 and 65–70 kDa, respectively, in both macrophage and renal fibroblast secretomes (Fig. 9a). But their levels in the fibroblast secretome were obviously greater than those in the macrophage secretome (Fig. 9a). Using Formula 3 to subtract macrophage-derived MMP-9 and MMP-2 from their total levels, the absolute fibroblast MMP-9 (Fig. 9b and Supplementary Data 6) and MMP-2 (Fig. 9c and Supplementary Data 7) levels were significantly greater in the COM-MP secretome-treated BHK-21 cells compared with the untreated and Ctrl-MP secretome-treated fibroblasts. Taken together, all these data indicate that the COM-MP secretome triggered transformation of renal fibroblast into its active form, myofibroblast.

## Discussion

Renal fibrosis, such as glomerulosclerosis and tubulointerstitial fibrosis, is a characteristic feature of chronic kidney disease (CKD) and is associated with several renal disorders, including kidney stone disease^38–40^. Such association has been explored in many previous studies, but mechanisms that drive the kidney with stone into fibrosis remain unclear. In general, renal fibrosis is resulted from tissue injury and inflammation, which are related to infiltrating leukocytes, particularly macrophage^41,42^. Tissue macrophage is one of the innate immune cells that plays important roles in recognition, phagocytosis, and elimination of pathogens, cellular debris and toxic materials^43,44^. Increasing evidence has suggested that macrophage plays crucial roles in pathogenic mechanisms of various kidney diseases by promoting inflammation-mediated fibrosis via their secretory products, including cytokines, growth factors, enzymes and matrix proteins^45–47^.

Interestingly, many previous studies have shown the presence of macrophages surrounding COM crystals in human and animal renal interstitial tissues^14–16^. There have been several attempts to explore the correlation between kidney stone disease and renal inflammation as well as fibrosis. It has been reported that COM crystal-exposed macrophages (COM-MP) can release transforming growth factor-β1 (TGF-β1), one of the proinflammatory cytokines, to induce epithelial-mesenchymal transition (EMT) of renal tubular epithelial cells^48,49^. These cells undergoing EMT finally transform into myofibroblasts, which play important roles in renal fibrogenesis^50^. Although renal myofibroblasts can be differentiated from tubular epithelial cells, they are derived mainly from residential renal fibroblasts^28–30^. Activation of renal fibroblasts to myofibroblasts can be induced by many cytokines, such as TGF-β1, platelet-derived growth factor (PDGF), nuclear factor kappa B inhibitor zeta (NF-κBIZ) and galectin-3, which are secreted from macrophages in response to various stimuli, including bacterial infection^51^, lipopolysaccharide (LPS) exposure^52^, and hypoxic condition^53^.

In this study, we hypothesized that COM-MP might contribute to kidney stone-induced renal fibrogenesis by secreting the fibrotic factors that trigger transformation of renal fibroblasts into myofibroblasts. We first defined the optimal condition to collect and to study secretome derived from the COM-MP under the serum-free culture condition. Note that the dosage of COM crystals used in the present study (100 µg/ml) was the non-toxic dose following the experimental findings reported in our previous studies^54–56^, whereas using the higher dose was related to obvious (unwanted) cytotoxicity^54,57,58^. The data showed that 16-h incubation of macrophages with COM crystals was the optimal time-point for harvesting and investigating their secretome, consistent with the results obtained from previous secretome studies on macrophages and monocytes exposed to COM crystals^20,59^.

Previous studies have demonstrated that COM-MP secrete exosomal proteins that play roles in immune response^20,60^. To provide more information, our present study has shown the correlation between changes in macrophage secretome induced by COM crystals and their roles in fibrogenesis. From a total of 598 secretory proteins, label-free quantitative proteomics analysis using nanoLC-ESI-LTQ-Orbitrap MS/MS identified 23 significantly altered proteins in the COM-MP secretome compared with the Ctrl-MP secretome. These significantly altered secretory proteins were involved mainly in inflammatory response (consistent with the previous studies^20,60^) and fibroblast activation (a novel information to be further explored).

Renal fibroblasts quiescently reside in renal interstitium and play an essential role in ECM homeostasis for maintaining the kidney ultrastructure and function^61,62^. During fibrogenesis, the quiescent fibroblasts are activated and display a spindle-shaped, mesenchymal phenotype, known as myofibroblasts^63,64^. In the present study, we found that BHK-21 cells treated with COM-MP secretome were more spindle-shaped and had greater spindle index, indicating that they underwent activation into myofibroblasts. These data were consistent with a previous study showing that BHK-21 renal fibroblasts exposed to hypoxia underwent myofibroblast transformation, in which the cells were elongated together with increasing spindle index^65^.

The activation of renal fibroblasts is also characterized by up-regulations of α-SMA and F-actin incorporated into the stress fibers^66–68^. During the transition of fibroblasts from quiescent state to their activated form, up-regulations of α-SMA and F-actin have been reported to promote myofibroblast contractile activity^67,69^. Several lines of evidence have indicated that α-SMA-associated contraction is involved in ECM organization^67,70,71^. The up-regulation of α-SMA is also important for myofibroblast focal adhesion, maturation and adhesive capability^72,73^. In tubulointerstitial renal fibrosis, TGF-β1 enhances α-SMA expression in fibroblasts to form stress fibers and focal adhesion, resulting in the fibrotic tissue contraction^74,75^. Our results showed the increased levels of both α-SMA and F-actin in the COM-MP secretome-treated BHK-21 cells, consistent with the myofibroblast features with up-regulations of α-SMA and F-actin at both transcript and protein levels in the hypoxia condition^65^.

The excessive accumulation of ECM is another important feature of tissue or renal fibrosis. Several previous studies have reported the increases of main ECM components, including collagens, fibronectin and proteoglycans in renal interstitial fibrotic tissues^76,77^. In addition, in vitro studies have demonstrated that α-SMA-expressing renal fibroblasts promote the synthesis of ECM components, including collagen type I and fibronectin^65,78^. In concordance, our present study revealed the increase in fibronectin in the COM-MP secretome-treated BHK-21 cells, indicating the fibrogenesis features of renal myofibroblasts.

In addition to excessive ECM synthesis, degradation and rearrangement of ECM components is crucial for renal fibrogenesis. The ECM remodeling requires several proteinases, especially matrix metalloproteinases (MMPs)^79^. Herein, we found the increases in MMP-9 and MMP-2 activities in the fibroblasts activated by COM-MP secretome. MMP-9 and MMP-2 possess strong gelatinase but weak collagenase activities. These MMPs play primary role in second step of collagen degradation to break down collagen molecules that have been denatured or cleaved by collagenases^80^. In renal fibrosis, there is increasing evidence demonstrating the roles for MMP-9 and MMP-2 in degradation of denatured collagens, fibronectin, laminin, elastin, and vitronectin^81,82^. They are also involved in the ECM remodeling^83,84^. Moreover, MMP-9 and MMP-2 have been suggested to induce fibrosis through TGF-β1 (a crucial mediator of fibrogenesis) and collagen-degrading proteases^83,84^. Since the increase in MMP-9 activity was more striking than that of MMP-2 in our present study, it was thus plausible that MMP-9 was more predominant than MMP-2 in driving renal fibrosis mediated by COM-MP secretome.

In summary, we report herein alterations in secretome from macrophages exposed to COM crystals. Functional annotation and protein-protein interactions network analysis indicated that they play roles in inflammatory response and fibroblast activation. Functional validation revealed that the COM-MP secretome induced renal fibroblast activation as demonstrated by the increased levels of the fibroblast activation markers, including spindle index, α-SMA and F-actin. In addition, the COM-MP secretome-treated fibroblasts had increased levels of the fibrotic factors, including fibronectin, MMP-9 and MMP-2. Taken together, these data indicate that macrophages exposed to COM crystals play important roles in renal fibrosis by secretome-induced activation of residential renal fibroblasts to myofibroblasts, the active form that is crucial for renal fibrogenesis.

## Materials and methods

### Cell lines and culture

Human monocyte-derived cell line (U937) (ATCC; Manassas, VA) and baby hamster kidney fibroblast cell line (BHK-21) (ATCC) were cultured in RPMI 1640 medium and Eagle’s minimum essential medium (Gibco; Grand Island, NY), respectively. These cells were grown in corresponding complete growth medium supplemented with 10% (v/v) heat-inactivated fetal bovine serum (Gibco), 60 U/ml penicillin G (Sigma-Aldrich; St. Louis, MO), and 60 µg/ml streptomycin (Sigma-Aldrich) in a humidified incubator at 37 °C with 5% CO_2_.

### COM crystal preparation

COM crystals were generated using the protocol established previously^85,86^. Briefly, 10 mM CaCl_2_·2H_2_O in a buffer containing 10 mM Tris-HCl and 90 mM NaCl (pH 7.4) was mixed 1:1 (v/v) with 1.0 mM Na_2_C_2_O_4_ in the same buffer to make their final concentrations at 5 mM and 0.5 mM, respectively. The mixture was incubated at 25 °C overnight and the COM crystals were harvested by a centrifugation at 2,000 × *g* for 5 min. The crystals were washed three times with methanol and air-dried overnight at 25 °C. The typical morphology of COM crystal was confirmed under an inverted phase-contrast light microscope (Eclipse Ti-S) (Nikon; Tokyo, Japan). The crystals were decontaminated by UV light radiation for 30 min before exposure to the cells.

### Differentiation of U937 monocytes into macrophages and COM crystal treatment

Macrophages were derived from U937 monocytic cells using phorbol 12-myristate 13-acetate (PMA) (Fluka; St. Loius, MO) for differentiation as previously described^87,88^. Briefly, U937 cells at a density of 5 × 10^5^ cells/ml were seeded in each well of the 6-well plate (Corning Inc.; Corning, NY) and treated with 100 ng/ml PMA for 48 h (induction phase). The cells were then vigorously washed three times with ice-cold PBS to remove PMA and non-adherent cells, whereas the adherent cells were further maintained as mentioned above for 48 h (recovery phase). The complete growth medium was refreshed daily. The characteristics of macrophages were observed under an inverted phase-contrast microscope (Eclipse Ti-S) as previously described^87,88^.

After 48-h recovery, the complete medium was removed followed by three washes with PBS. The adherent macrophages were then incubated in serum-free medium with or without 100 µg/ml COM crystals. At 16, 24 and 48-h post-incubation, the cells were subjected to cell death analysis as follows.

### Quantitative analysis of cell death by flow cytometry

Flow cytometry was performed to quantitate cell death as previously described^59^. At 16, 24 and 48-h after COM exposure as mentioned above, macrophages were trypsinized with 0.1% trypsin in 2.5 mM EDTA/PBS. The cells were harvested by a centrifugation at 500 × *g* and 4 °C for 5 min and washed twice with ice-cold PBS. After PBS removal, the cell pellets were resuspended with annexin V buffer (10 mM HEPES, 140 mM NaCl and 2.5 mM CaCl_2_·2H_2_O; pH 7.4) and further incubated with FITC-labeled annexin V (BD Biosciences; San Jose, CA) on ice in the dark for 15 min. Propidium iodide (BD Biosciences) at the final concentration of 0.2 µg/ml was added into the cell suspension prior to analysis using a flow cytometer (BD Accuri C6) (BD Biosciences). Percentage of cell death was calculated using the following formula:

1 $${{{{{\mathrm{Cell}}}}}}\,{{{{{\mathrm{death}}}}}}\,({ \% })=({{{{{\mathrm{Numbers}}}}}}\,{{{{{\mathrm{of}}}}}}\,{{{{{\mathrm{dead}}}}}}\,{{{{{\mathrm{cells/Total}}}}}}\,{{{{{\mathrm{number}}}}}}\,{{{{{\mathrm{of}}}}}}\,{{{{{\mathrm{all}}}}}}\,{{{{{\mathrm{cells}}}}}})\,\times {100}$$

### Collection and preparation of macrophage secretome

After 16-h post-incubation with or without crystals in serum-free medium, the culture supernatant was collected from the control macrophages (Ctrl-MP) and the COM-exposed macrophages (COM-MP). Cell debris and particulate matters were removed by a centrifugation at 2000 × *g* and 4 °C for 5 min. The clear supernatant containing a set of secretory proteins (secretome) was then subjected to quantitative proteomics and used for renal fibroblast treatment as follows.

### In-solution tryptic digestion by filter-aided sample preparation (FASP) method

Equal amount of total protein derived from each sample was digested by trypsin according to FASP protocol^89,90^. Briefly, the protein mixture in SDT buffer was reduced by heating at 95 °C for 5 min. After cooling down at 25 °C, the sample was transferred to an Omega Nanosep 10 K device (Pall Corporation; Port Washington, NY), added with 200 µl of 8 M urea in 100 mM Tris-HCl (pH 8.5), and then centrifuged at 14,000 × *g* and 25 °C for 15 min. This buffer exchange step was repeated one more cycle. The recovered proteins were then alkylated with 100 µl of 50 mM iodoacetamide in 8 M urea/100 mM Tris-HCl (pH 8.5) at 25 °C in the dark using a ThermoMixer C (Eppendorf; Hauppauge, NY) for 20 min. Thereafter, buffer exchange was performed twice by centrifugation at 14,000 × *g* and 25 °C for 15 min each using 200 µl of 8 M urea/100 mM Tris-HCl (pH 8.5). The proteins were then finally exchanged into 50 mM NH_4_HCO_3_ and then digested with sequencing grade modified trypsin (Promega; Madison, WI) in 50 mM NH_4_HCO_3_ at a ratio of 1:50 (w/w) trypsin/protein at 37 °C for 16 h in a ThermoMixer C. The digested peptides were collected by transferring the filter unit to a new collection tube and centrifuged at 14,000 × *g* at 25 °C for 15 min. Trypsin activity was then stopped by adding 10 µl of 5% formic acid in 80% acetonitrile (ACN), and the digested peptides were dried by a vacuum concentrator (ScanVac; Lynge, Denmark). The peptides were finally resuspended in 0.1% formic acid prior to tandem mass spectrometry (MS/MS).

### nanoLC-ESI-LTQ-Orbitrap MS/MS

Three independent biological replicates were analyzed in each group and each sample was run in technical triplicates. Separation of the digested peptides was performed using EASY-nLC II (Thermo Scientific; Waltham, MA). Briefly, peptides were loaded from a cooled (7 °C) autosampler into an in-house, 3-cm-long pre-column containing 5-µm C18 resin (Dr.Maisch GmbH; Ammerbuch, Germany) and then to an in-house, 10-cm-long analytical column packed with 3-µm C18 resin (Dr. Maisch GmbH) using mobile phase A (0.1% formic acid). The peptides were then separated by mobile phase B (ACN/0.1% formic acid) gradient elution with four steps as follows: 2–9% for 15 min, 9–35% for 85 min, 35–95% for 20 min, and then 95% for 10 min at a flow rate of 200 nl/min. Peptide sequences were then analyzed by LTQ-Orbitrap-XL (Thermo Scientific) in positive mode with ESI nanosprayer ion source^91,92^.

Data were acquired in a collision-induced dissociation (CID) top-12 mode under the control of the Xcalibur 2.1.0 and LTQ Tune Plus 2.5.5 software (Thermo Scientific). The cycle of one full scan was performed at a resolution of 30,000 (300–2000 *m/z*) in the Orbitrap followed by 12 data-dependent MS/MS scans in the linear ion trap with enabled preview mode for FTMS master scan. The minimum signal threshold at 1 × 10^5^ was required for a precursor ion to be selected for further fragmentation. Accumulation target values of full MS and MS/MS scan were 5 × 10^5^ and 3 × 10^4^ ions, respectively. Singly charged ions and unassigned charge states were excluded for fragmentation. Helium was used as a collision gas and the normalized collision energy was set at 35%. The activation time was 30 ms for acquiring mass spectra. The duration of dynamic exclusion was 180 s.

### MS/MS spectral interpretation and quantitative analysis

The MS/MS raw spectra were deconvoluted and then extracted into output searchable*.mgf* files using Proteome Discoverer v.1.4.1.14 software (Thermo Scientific). Mascot software version 2.4.0 (Matrix Science; London, UK) was used to search MS/MS spectra against SwissProt database of humans with the following standard Mascot parameters for CID: Enzyme = trypsin, maximal number of missed cleavages = 1, peptide tolerance = ±2 ppm, MS/MS tolerance = ±0.2 Da, fixed modification = carbamidomethyl (C), variable modification = oxidation (M), charge states = 2+ and 3+, and decoy database on FDR <1%^93,94^. Quantitative data of each protein was obtained from averaging areas under curve (AUC) (or peak areas) of peptide precursor ion intensity of the three most abundant peptides identified from each protein. Note that background was subtracted from all peak areas.

### Bioinformatics and protein–protein interactions network analyses

Functional annotation and enrichment analysis of significantly altered proteins were performed using g:GOSt tool in g:Profiler web server (https://biit.cs.ut.ee/gprofiler/gost). Molecular interaction networks were visualized by Cytoscape version 3.7.2 (www.cytoscape.org/). In addition, all significantly altered proteins were analyzed for protein-protein interactions network using STRING tool version 11.0 (www.string-db.org).

### Treatment of BHK-21 renal fibroblasts with macrophage secretome

BHK-21 cells (2.5 × 10^4^ cells) were seeded in each well of the 24-well plate (Corning Inc.) and maintained in its complete growth medium overnight. Thereafter, the complete medium was removed and the cells were washed with PBS three times. The cells were then incubated in serum-free medium mixed 1:1 (v/v) with either Ctrl-MP or COM-MP clear culture supernatant. In parallel, the cells incubated in the fresh serum-free medium without culture supernatant served as the untreated control. After 24-h incubation, the BHK-21 cells were analyzed by the following assays.

### Morphology and spindle index of renal fibroblasts

Effects of Ctrl-MP secretome vs. COM-MP secretome on cell morphology were examined and imaged under an inverted phase-contrast light microscope (Eclipse Ti-S). Cell boundary was manually determined using NIS-Elements D V.4.11 software (Nikon; Tokyo, Japan), whereas length and width of each cell were automatically measured by this software. Spindle index^65,95^ was calculated from at least 100 cells in ≥10 random high-power fields (HPFs) of each sample by using the following formula:

2 $${{{{{\mathrm{Spindle}}}}}}\,{{{{{\mathrm{index}}}}}}={{{{{\mathrm{Length}}}}}}\,{{{{{\mathrm{of}}}}}}\,{{{{{\mathrm{each}}}}}}\,{{{{{\mathrm{cell/Width}}}}}}\,{{{{{\mathrm{of}}}}}}\,{{{{{\mathrm{each}}}}}}\,{{{{{\mathrm{cell}}}}}}$$

### Immunofluorescence staining of renal fibroblasts

The cells were grown on coverslips and treated as described above. Immunofluorescence staining was performed as described previously^96,97^. Briefly, the cells were washed twice with PBS and fixed with 4% paraformaldehyde in PBS at 25 °C for 15 min. The fixed cells were permeabilized with 0.1% Triton X-100 in PBS at 25 °C for 15 min and then washed with PBS. Thereafter, non-specific bindings were blocked with 1% BSA in PBS at 25 °C for 30 min. The cells were incubated with mouse monoclonal anti-α-SMA (Santa Cruz Biotechnology; Santa Cruz, CA) or mouse monoclonal anti-fibronectin antibody (Santa Cruz Biotechnology) (diluted 1:50 in 1% BSA/PBS) or phalloidin conjugated with Oregon Green 488 (Invitrogen; Eugene, OR) at 37 °C for 1 h. The cells were washed three times with PBS and further incubated with Alexa Flour 488-conjugated donkey anti-mouse IgG antibody (diluted 1:500 in 1% BSA/PBS) (Invitrogen) mixed with Hoechst dye (Sigma-Aldrich) (for nuclear staining) (diluted 1:1000 in 1% BSA/PBS) at 37 °C for 1 h. Finally, the coverslips were mounted onto glass slides using 50% glycerol in PBS and the fluorescence images were captured under a fluorescence microscope (Eclipse 80i) (Nikon). Quantitative data were obtained and analyzed from at least 100 cells in ≥10 random HPFs for each sample using NIS-Elements D V.4.11 (Nikon).

### Measurement of renal fibroblast matrix metalloproteinase-9 and -2 (MMP-9 and MMP-2) activities

The activities of MMP-9 and MMP-2 were measured using the gelatin zymogram assay as described previously^98,99^. Briefly, the serum-free culture supernatant was collected from BHK-21 cells after treatment without or with Ctrl-MP secretome or COM-MP secretome for 24 h. Cellular debris was removed by centrifugation at 1000 × *g* for 5 min. Equal volume of the samples (10 µl/sample) was mixed with a non-reducing buffer (50 mM Tris-HCl, 2% SDS, and 10% glycerol) and then resolved in 10% polyacrylamide gel (Bio-Rad; Berkeley, CA) co-polymerized with 0.1% (w/v) gelatin (Sigma-Aldrich). Thereafter, the gel was washed three times with a renaturation buffer (2.5% Triton X-100) at 25 °C for 15 min followed by other three washes with an activation buffer (50 mM Tris-HCl, 150 mM NaCl, and 10 mM CaCl_2_; pH 8.0) and incubated with the activation buffer at 37 °C overnight. The gel was then stained with 0.05% Coomassie Blue G-250 (Sigma-Aldrich) for 2 h and de-stained with distilled water for another 2 h. Finally, the transparent bands reflecting the MMP-9 and MMP-2 activities were quantified using ImageQuant TL software (GE Healthcare; Uppsala, Sweden).

Because levels of MMP-9 and MMP-2 in the BHK-21 cell culture supernatant might be derived from both renal fibroblasts and macrophage secretome, their levels were also measured in the Ctrl-MP and COM-MP culture supernatants (to subtract their levels in the corresponding BHK-21 culture supernatants). The absolute fibroblast MMP-9 or MMP-2 level was calculated using the following formula:

3 $${{{{{\mathrm{Absolute}}}}}}\,{{{{{\mathrm{fibroblast}}}}}}\,{{{{{\mathrm{MMP(X)}}}}}}\,{{{{{\mathrm{level}}}}}}=	\,{{{{{\mathrm{MMP(X)}}}}}}\,{{{{{\mathrm{level}}}}}}\,{{{{{\mathrm{in}}}}}}\,{{{{{\mathrm{fibroblast}}}}}}\,{{{{{\mathrm{culture}}}}}}\,{{{{{\mathrm{supernatant}}}}}}\\ 	\,\hskip -2.8pt{{-}}\,{{{{{\mathrm{MMP(X)}}}}}}\,{{{{{\mathrm{level}}}}}}\,{{{{{\mathrm{in}}}}}}\,{{{{{\mathrm{corresponding}}}}}}\,{{{{{\mathrm{macrophage}}}}}}\,{{{{{\mathrm{secretome}}}}}}$$

Where X represented each form of MMPs, i.e., MMP-9 and MMP-2.

### Statistics and reproducibility

All the quantitative data are reported as mean ± SEM of the measurements done in three independent experiments using different biological replicates. Comparisons between two groups were done by unpaired Student’s *t*-test, whereas multiple comparisons were performed using one-way ANOVA with Tukey’s post-hoc test. *P*-values less than 0.05 were considered statistically significant.

### Reporting summary

Further information on research design is available in the Nature Research Reporting Summary linked to this article.

## Supplementary information

Supplementary Data 1

Supplementary Data 2

Supplementary Data 3

Supplementary Data 4

Supplementary Data 5

Supplementary Data 6

Supplementary Data 7

Reporting Summary

Description of Additional Supplementary Files

## Data Availability

All data generated or analyzed during this study are included in this published article and supplementary files. The source data for Figs. 1b, 5b, 6b, 7b, 8b, 9b, and 9c are provided in Supplementary Data 1–7, respectively. The mass spectrometry proteomics data have been deposited to the ProteomeXchange Consortium (http://www.proteomexchange.org/) via the PRIDE (https://www.ebi.ac.uk/pride/) partner repository with the dataset identifier PXD027039 and 10.6019/PXD027039.
