# Supplementary material for: Effects of secretome derived from macrophages exposed to calcium oxalate crystals on renal fibroblast activation
Source: Commun Biol. 2021 Aug 11;4:959. doi: 10.1038/s42003-021-02479-2 (PMC8358035; doi:10.1038/s42003-021-02479-2)
Supplement: Supplementary file 8 — Reporting Summary [file 42003_2021_2479_MOESM8_ESM.pdf]

nature research

Corresponding author(s): Prof. Visith Thongboonkerd

Last updated by author(s): May 1, 2021

Reporting Summary

Nature Research wishes to improve the reproducibility of the work that we publish. This form provides structure for consistency and transparency in reporting. For further information on Nature Research policies, see our Editorial Policies and the Editorial Policy Checklist.

Statistics

For all statistical analyses, confirm that the following items are present in the figure legend, table legend, main text, or Methods section.

n/a	Confirmed	
☐	☒	The exact sample size (n) for each experimental group/condition, given as a discrete number and unit of measurement
☐	☒	A statement on whether measurements were taken from distinct samples or whether the same sample was measured repeatedly
☐	☒	The statistical test(s) used AND whether they are one- or two-sided<br>*Only common tests should be described solely by name; describe more complex techniques in the Methods section.*
☒	☐	A description of all covariates tested
☐	☒	A description of any assumptions or corrections, such as tests of normality and adjustment for multiple comparisons
☐	☒	A full description of the statistical parameters including central tendency (e.g. means) or other basic estimates (e.g. regression coefficient) AND variation (e.g. standard deviation) or associated estimates of uncertainty (e.g. confidence intervals)
☐	☒	For null hypothesis testing, the test statistic (e.g. F, t, r) with confidence intervals, effect sizes, degrees of freedom and P value noted<br>*Give P values as exact values whenever suitable.*
☒	☐	For Bayesian analysis, information on the choice of priors and Markov chain Monte Carlo settings
☒	☐	For hierarchical and complex designs, identification of the appropriate level for tests and full reporting of outcomes
☒	☐	Estimates of effect sizes (e.g. Cohen's d, Pearson's r), indicating how they were calculated

Our web collection on statistics for biologists contains articles on many of the points above.

Software and code

Policy information about availability of computer code

Data collection	- Not applicable.
Data analysis	- Not applicable.

For manuscripts utilizing custom algorithms or software that are central to the research but not yet described in published literature, software must be made available to editors and reviewers. We strongly encourage code deposition in a community repository (e.g. GitHub). See the Nature Research guidelines for submitting code & software for further information.

Data

Policy information about availability of data

All manuscripts must include a data availability statement. This statement should provide the following information, where applicable:

- Accession codes, unique identifiers, or web links for publicly available datasets
- A list of figures that have associated raw data
- A description of any restrictions on data availability

All data generated or analyzed during this study are included in this published article and supplementary files. The source data for Fig. 2b, 5b, 6b, 7b, 8b, 9b, and 9c are provided in Supplementary Data 1 – 7, respectively. The mass spectrometry proteomics data have been deposited to the ProteomeXchange Consortium (http://www.proteomexchange.org/) via the PRIDE (https://www.ebi.ac.uk/pride/) partner repository with the dataset identifier PXD027039 and 10.6019/PXD027039

Field-specific reporting

Please select the one below that is the best fit for your research. If you are not sure, read the appropriate sections before making your selection.

- [x] Life sciences
- [ ] Behavioural & social sciences
- [ ] Ecological, evolutionary & environmental sciences

For a reference copy of the document with all sections, see nature.com/documents/nr-reporting-summary-flat.pdf

Life sciences study design

All studies must disclose on these points even when the disclosure is negative.

Sample size	Cell lines were used and thus required no sample size calculation.
Data exclusions	Data were not excluded from analysis.
Replication	All quantitative data were obtained from three independent experiments.
Randomization	Spindle index and immunofluorescence intensities were measured from at least 100 cells in ≥10 random fields.
Blinding	Blinding was not possible as experimental conditions were evident from the image data. Quantifications were performed using computational pipeline applied equally to all conditions and replicates for a given probe. Thresholds for detecting puncta were chosen for each probe based on graphs with objective properties that appeared indistinguishable across conditions.

Reporting for specific materials, systems and methods

We require information from authors about some types of materials, experimental systems and methods used in many studies. Here, indicate whether each material, system or method listed is relevant to your study. If you are not sure if a list item applies to your research, read the appropriate section before selecting a response.

Materials & experimental systems

n/a	Involved in the study	
[ ]	[x]	Antibodies
[ ]	[x]	Eukaryotic cell lines
[x]	[ ]	Palaeontology and archaeology
[x]	[ ]	Animals and other organisms
[x]	[ ]	Human research participants
[x]	[ ]	Clinical data
[x]	[ ]	Dual use research of concern

Methods

n/a	Involved in the study	
[x]	[ ]	ChIP-seq
[x]	[ ]	Flow cytometry
[x]	[ ]	MRI-based neuroimaging

Antibodies

Antibodies used	Primary antibodies:<br>-Mouse monoclonal anti-α-SMA (Santa Cruz Biotechnology; Santa Cruz, CA)<br>-Mouse monoclonal anti-fibronectin (Santa Cruz Biotechnology)<br>Secondary antibody:<br>-Alexa Flour 488-conjugated donkey anti-mouse IgG antibody (Invitrogen)
Validation	All antibodies were validated and datasheets are available.

Eukaryotic cell lines

Policy information about cell lines

Cell line source(s)	-U937 (human, ATCC)<br>-BHK-2 (hamster, ATCC)
Authentication	Cell line authentication was initially performed by ATCC. In addition, each of them had distinct morphology.
Mycoplasma contamination	Cell lines were periodically tested negative for Mycoplasma and there were no signs of Mycoplasma contamination observed.
Commonly misidentified lines (See ICLAC register)	Not applicable.
