# Supplementary material for: Effects of secretome derived from macrophages exposed to calcium oxalate crystals on renal fibroblast activation
Source: Commun Biol. 2021 Aug 11;4:959. doi: 10.1038/s42003-021-02479-2 (PMC8358035; doi:10.1038/s42003-021-02479-2)
Supplement: Supplementary file 9 — Description of Additional Supplementary Files [file 42003_2021_2479_MOESM9_ESM.pdf]

Description of Additional Supplementary Files

Files: Supplementary Data 1-7

Description:

The source data for Fig. 1b, 5b, 6b, 7b, 8b, 9b, and 9c are provided in Supplementary Data 1 – 7, respectively.

Supplementary Data 1: Source data for Fig. 1b

Supplementary Data 2: Source data for Fig. 5b

Supplementary Data 3: Source data for Fig. 6b

Supplementary Data 4: Source data for Fig. 7b

Supplementary Data 5: Source data for Fig. 8b

Supplementary Data 6: Source data for Fig. 9b

Supplementary Data 7: Source data for Fig. 9c
